# Toward Autonomous Prostate Cancer Clinical Significance Determination from Spectral/Statistics Features in Bi-Parametric MRI

**DOI:** 10.3390/cancers18152473

**Published:** 2026-08-01

**Authors:** Rulon Mayer, Yuan Yuan, Jayaram Udupa, Baris Turkbey, Charles B. Simone

**Affiliations:** 1 Oncoscore, Garrett Park, MD 20896, USA; 2 The University of Sydney, Sydney, NSW 2050, Australia; y.yuan@sydney.edu.au; 3 Perelman School of Medicine, University of Pennsylvania, Philadelphia, PA 19104, USA; jay@pennmedicine.upenn.edu; 4 National Institutes of Health, Bethesda, MD 20892, USA; 5 New York Proton Center, New York, NY 10035, USA; csimone@nyproton.com

**Keywords:** prostate cancer, multi-parametric MRI, artificial intelligence, color vision, bi-parametric MRI

## Abstract

**Background/Objectives**: Deciding between active surveillance and treatment for prostate cancer patients often requires accurate risk assessment of prostate tumors detected with multi-parametric MRI. Conventionally, radiologists visually inspect MRI and use scoring procedures such as PI-RADS to help assess the scans. More recently, artificial intelligence (AI) applied to MRI has allowed for supplementation and is complementary to clinical assessment. However, AI is computationally expensive and severely saps scarce energy and water resources and requires special processing components, requiring alternate approaches that require less computation and fewer resources. The novel, simpler spectral/statistics approach that mimics color vision was previously successfully applied in a number of retrospective pilot studies of bi-parametric MRI of prostate cancer. The novel approach needs far fewer resources, is less computationally intensive, and is simpler than artificial intelligence to evaluate prostate tumors. However, these earlier spectral/statistics pilot studies required intervention by an analyst and too much time for implementation in future large patient studies that are needed to validate the novel approach. This retrospective pilot study further developed, applied, and tested new automation tools to expedite simpler spectral statistical techniques that need fewer resources to evaluate prostate tumors on multi-parametric MRI. **Methods**: Automated spatial registration, automated prostate organ segmentation, automated blob generation and selection for spectral signatures derived from the apparent diffusion coefficient, high-B-value DWI, and T2 MRI were performed on 76 consecutive patients in the PI-CAI cohort in this retrospective pilot study. The signal-to-clutter ratio (SCR) was computed using target signatures and the processed statistical metrics of the registered prostate bi-parametric MRI. The processed SCR, spectral/spatial features of blobs and clinical metrics predict clinically significant prostate cancer using multivariate logistic regression. The proposed method was assessed using the area under the curve (AUC) from the receiver operating characteristic curve. **Results**: AUC values of >0.90 were achieved by combining the SCR with blob and clinical metrics. Increasing the number of non-congruent, independent variables resulted in higher AUC scores. Restricting analysis to blob volumes > 0.1 cm^3^ achieved higher AUC values. The additional total savings in time by applying the new automation tools reduced the processing time by 80 to 170 min for 10 patients. Implementing the new automation tools resulted in an overall processing time of 40 to 80 min per 10 patients. **Conclusions**: Automating the spectral/statistics approach resulted in AUCs not inferior to those obtained from AI. The automation achieved sufficiently high AUCs and also reduced processing times, warranting future assessments in large patient cohorts.

## Simple Summary

Deciding between active surveillance and treatment for prostate cancer patients often requires accurate assessment of prostate tumors. Independent fast quantitative analysis may provide a check and support for radiologists who conventionally visually inspect MRI and may follow protocols such as PI-RADS. Artificial intelligence is increasingly being applied to help clinical assessment of prostate tumors detected with multi-parametric MRI, just as in other fields. However, implementation of artificial intelligence and deep learning is computationally intensive, requires fast, high-end processing boards and, therefore, uses considerable energy and water resources for cooling. A less intensive, less wasteful, cheaper, and less environmentally destructive assessment approach is needed. The simpler spectral/statistics approach that mimics color vision was previously successfully applied in retrospective pilot studies of bi-parametric MRI of prostate cancer. The novel approach needs far fewer resources, is less computationally intensive, and is simpler than artificial intelligence to evaluate prostate tumors. However, these earlier spectral/statistics pilot studies required the intervention of an analyst and too much time for implementation in future large patient studies that are needed to validate the novel approach. This study developed, applied, and tested new automation tools to expedite the spectral/statistics approach. Automating the novel approach resulted in sufficiently high AUCs and a reduction in processing time, warranting future applications in large patient cohorts.

## 1. Introduction

Determining whether a prostate tumor will grow, spread, metastasize or remain stable plays a critical role in the clinical management of prostate cancer [1]. The prostate cancer’s stage determines whether the cancer needs to be treated or actively surveyed and observed. Multi-parametric MRI (MP-MRI) scanning [2], which includes images showing the time evolution of contrast material injected into the patient and structural and diffusion modalities, is one of the principal tools used to assess prostate tumors. Injecting contrast material reveals the tumor vasculature and provides valuable information regarding the prostate tumor. However, scanning protocols are increasingly foregoing contrast scans, and patients are instead scanned with two pulse sequences—diffusion-weighted imaging (DWI), specifically the apparent diffusion coefficient (ADC), and high b-value (HBV), along with the anatomic T2 imaging, to reduce patient inconvenience and expedite clinical flow—which is known as bi-parametric (BP) MRI [3,4,5,6,7,8,9]. A tumor may distinguish itself from normal prostate due to the tumor’s higher cellular density, which results in diffusion restriction, and this physiology can appear in diffusion-related MRI [10,11,12,13,14]. Conventionally, clinicians visually inspect the prostate MRI scans and deduce the prostate cancer condition by following a protocol such as PI-RADS [15,16,17].

Recently [18,19,20,21,22,23,24], research studies tested to see if applying artificial intelligence (AI) to bi-parametric MRI and quantitative assessments could potentially supplement and/or complement clinical evaluation. AI generates spatial features in individual images and uses the morphology and neuronal modeling to help predict the clinical condition of prostate cancer and does not exploit the spectral nature of the data. Although promising, AI approaches employ high-end processing boards whose operation can strain local energy and water resources [25,26,27]. Alternative simpler spectral/statistical approaches [28,29,30,31,32] adapted from remote sensing and biology, such as color vision, are far less computationally intensive, less demanding of local resources and destructive to the environment, and also generate additional, diverse, non-congruent features leading to better tumor evaluations. In addition, end-to-end AI models, being black boxes, lack interpretability and can be difficult to trust by clinicians. In contrast, spectral statistical features offer easier interpretability.

Following extensive training and testing [18,19,20,21,22,23,24], AI conventionally generates a large number of spatial features, such as textures, filters out irrelevant ones, and then combines them through emulating neuronal processing and structures to predict the tumor status. The neuronal model requires a large number of parameters and substantial resources. In contrast [28,29,30,31,32], spectral statistical processing follows color vision by replicating spatial registration for color cones (red, green, and blue) and substituting with the bi-parametric MRI modalities, namely, apparent diffusion coefficient (ADC), high-B-value DWI, and T2. The spectral/statistical model requires far fewer parameters, and the spectral features follow analytic approaches rather than being derived from extensive training.

The spectral/statistical approach is relatively new. It has been tested on datasets such as a 26-patient multispectral MRI cohort that included kinetic images derived from dynamic contrast-enhanced MRI as well as standard MRI (ADC, HBV, and T2) [28] and clinical data, and also in a 42-patient cohort composed of bi-parametric MRI [29,30,31,32] without contrast-enhanced images. These pilot studies showed promise and achieved comparable performance with AI [32]. However, the studies were limited by the small number of patients in the analyses. In addition, significant components (organ segmentation, registration, and spectral signature extraction) in those studies required manual intervention, greatly increasing the time to process the data. The manual intervention that requires extra analysis time prevents future studies involving a large number of patients that could validate earlier pilot studies. A previous study [31] automated spectral signature generation, a part of the overall spectral signature prediction of prostate cancer significance. In this study, new automated spectral/statistical approaches enable and expedite the analysis for future testing in a large cohort of patients. Specifically, this study added an automated spatial registration of the individual BP-MRI components (ADC, HBV, and T2) and also autonomous prostate organ masking. This current, retrospective, pilot study does not and cannot definitively demonstrate that spectral/statistical methods are superior or not inferior to AI due to the limited number of patients. Instead, this study shows the feasibility of applying spectral/statistical methods to a larger number of patients within a reasonable time, achieving sufficient accuracy in classifying prostate cancer status, and, therefore, merits further studies on a much larger patient cohort.

## 2. Materials and Methods

### 2.1. Overview

**Figure 1:**
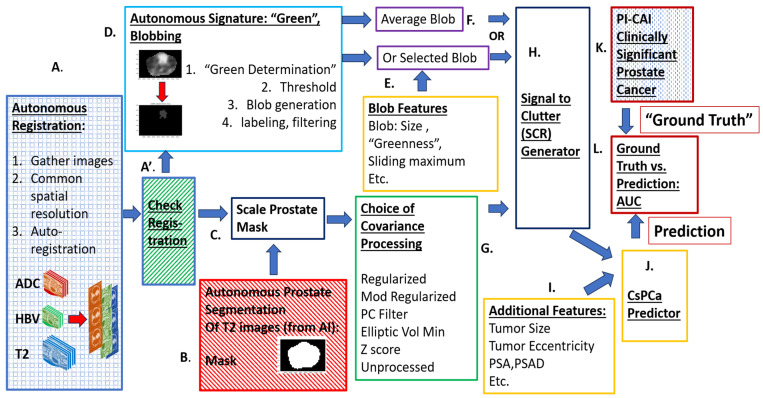
Overall schematic showing procedure to generate CsPCa prediction. (**A**) Spatial registration, (**A′**) manual registration check, (**B**) AI masking, (**C**) mask scale, (**D**) blobbing, (**E**) select blob based on blob features, (**F**) select or average SCR, (**G**) process background covariance matrix, (**H**) compute SCR, (**I**) clinical features, (**J**) predict CsPCa, and compare with (**K**) PI-CAI ground truth, using (**L**). AUC metrics computation to assess CsPCa prediction. Only the highlighted green box in Table 1 (labeled (**A′**)) is not automated but serves as a check for the spatial registration. The only contribution from AI (prostate masking, labeled (**B**)) is highlighted in red. The only non-image-related component is the “ground truth” highlighted in blue that is provided by the biopsies and labeled (**K**).

Figure 1 displays, schematically, the overall process for computing a spectral feature, the signal-to-clutter ratio (SCR), and its role in predicting the clinical significance of prostate cancer (CsPCa). The spatial registration, SCR generation, and CsPCa prediction proceed in certain discrete steps. Almost all steps were automated and, therefore, expedited in this study, unlike in previous efforts. This study added automated spatial registration of the individual BP-MRI components (ADC, HBV, and T2) and prostate organ masking. Only the highlighted green box in Table 1 (labeled A′) is not automated but serves as a check for the automated spatial registration. The only contribution from AI (prostate masking, labeled B) is highlighted in red. The only non-image-related component is the “ground truth” highlighted in blue, provided by the biopsies and labeled K. Using a custom code written in python 3.11 on a 13th-Gen Intel ^®^ Core with 16 GB of RAM, the spatially registered spectral hypercubes (A) were first assembled from the stored apparent diffusion coefficient (ADC), high-B value (HBV), and T2 data from the PI-CAI archive. The spatial registration was visually checked and corrected (A’) by applying small transverse translations and/or slice adjustments in the axial direction when needed. Hypercubes are spatially registered cubes of ADC, HBV, and T2, based on individual slices, and were stitched together to depict the entire scanned region. AI segmentation identified the prostate organ from the T2 image (B) and was identified as the prostate organ mask (B). The mask image was subsequently spatially rescaled (C) to match the ADC, HBV and hypercube transverse spatial resolution. The “greenest” parts (D) of the hypercube were found by computing the ratio of scaled HBV/(ADC + HBV + T2) and then accepting “green” voxel values that exceed a threshold, thereby identifying regions with the highest “green” values or likelihood of purported tumor presence. The “greenest” regions were blobbed and spatially filtered. Based on blob size, position, “greenness”, etc., the appropriate blob was selected (E). The spectral signature was taken from the selected blob based on the center of mass (F). The SCR was computed (H) based on the spectral signature and processing of the statistics (such as regularization and filtering) (G) of the covariance matrix for the prostate (or background). The SCR was combined with clinical factors (I), such as prostate serum antigen (psa), prostate volume, etc., to predict the CsPCa. The “ground-truth” CsPCa (K) in the PI-CAI data provided a benchmark for the area-under-the-curve (AUC) calculations (L).

Details regarding the method are summarized in the following Methods subsections. Further details are discussed and cited in the references.

### 2.2. Study Design and Population

The PI-CAI challenge [24] stores an annotated multi-center, multi-vendor publicly available dataset of 1500 bi-parametric MRI exams. The publicly available PI-CAI data collection [24] only includes bi-parametric MRI, specifically ADC, HBV, and T2 sequences. This collection includes clinical and acquisition variables. A variety of histopathology techniques were applied to the patients [24]. Most of the 1500 patients in the study had biopsies. Patients were scanned at a variety of centers using Siemens and Philips scanners. Clinically significant prostate cancer was identified for patients with Gleason or ISUP scores ≥ 2 [33].

Previously [30,31,32], 42 consecutive patients who had been biopsied in the PI-CAI database were assessed. In this dataset, 76 consecutive patients were examined. All patients exhibited biopsy-proven adenocarcinoma of the prostate, with a mean patient age of 64.1 years (range: 43 to 78 years), a mean PSA of 11.63 ng/mL (range: 0.1 to 81.95 ng/mL), a mean prostate volume of 68.5 cm^3^ (range: 19 to 279 cm^3^), a mean PSA density (PSAD) of 0.248 ng/mL/mL (range: 0.0034 to 4.3132 ng/mL/mL) and a mean ISUP grade of 0.763 (range: 0 to 5); 62 and 14 patients were confirmed as having clinically insignificant and significant prostate cancer, respectively. No restrictions on tumor location (peripheral, central, and transition) within the prostate were placed for this study. Informed consent was exempted due to the retrospective nature of this study. All patient data were anonymized.

### 2.3. Spatial Registration

**Figure 2:**
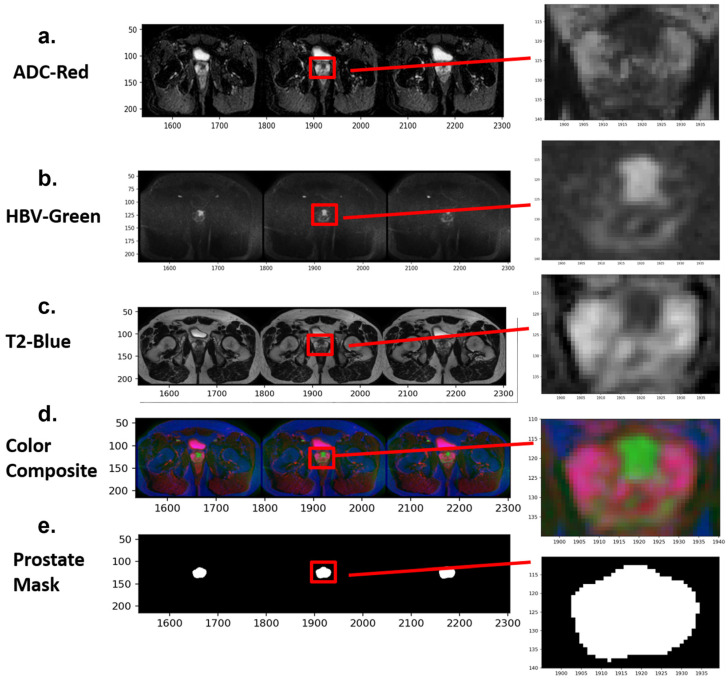
Depiction of three registered slices and the corresponding ADC (Red), HBV (Green), T2 (Blue), the color composite, and the prostate mask in (**a**), (**b**), (**c**), (**d**), (**e**), respectively. Zoomed sections of the prostate for a single slice, also shown in (**a**), (**b**), (**c**), (**d**), (**e**), respectively. The images were taken from Patient #10085 in the PI-CAI data collection.

Spatial-registered hypercube formation was previously described in [28,29,30,31,32] and is depicted and listed in Figure 1 (Panel A) and displayed in Figure 2. The following describes new automation procedures implemented for this study. This example was taken from patient #10085 in the PI-CAI data collection. All rescaling, re-slicing, and resampling were based on the spatial resolution, with slice positions listed in the header files. The ADC–HBV images have the lowest transverse spatial resolution and are, therefore, used as a reference for resampling the T2 image. Due to the prostate organ masking for T2, the T2 images were used as the reference for re-slicing the ADC–HBV slices in the axial direction. Two slicing options were applied. First, the ADC–HBV files were resampled based on their relative slice positions with respect to the T2 slice positions. Alternatively, the ADC–HBV files remained intact and were assigned to the closest T2 position. Transverse cropping was based on the images with the smaller field-of-view (usually the ADC–HBV). Both axial resampling methods ultimately yielded similar results in terms of predicting the CsPCa. The axial cropping was based on the T2 slice arrangement and also the presence of prostate organ masks. Three consecutive spatially registered, stitched segments for ADC (Figure 2a), HBV (Figure 2b), and T2 (Figure 2c) were combined into a color composite (Figure 2d) after assigning the colors red, green, and blue, respectively, to the images. The color composite highlights the tumor and also shows any evident misregistration among the images.

Due to the great variety of scanning techniques in the PI-CAI data collection [23,24,25,26,27], each patient was manually and visually checked, and small translations and slice adjustments were applied when necessary to achieve registration to the one-voxel level. Tumors, regions within the prostate, and prostate structures generally extend over many voxels, mitigating spatial misregistration at the voxel level.

### 2.4. Prostate Organ Masking and Scaling

Previously [28,29,30,31,32], the prostate organ mask was manually outlined using the ADC–HBV images. This study instead applied prostate organ masking to the T2 images using the segmentation techniques described by Yuan et al. [34] and depicted and listed in Figure 1 (Panel B). Yuan and colleagues created segmentation and classification models without manual labeling that were also self-supervised and generic. Extensive experiments demonstrated that Yuan’s models significantly outperformed learning from scratch and existing pre-trained 3D models. The high-achieving performance was due to the unified self-supervised learning framework that uses recurrent anatomy in medical images.

Yuan et al.’s mask images were resampled to match the transverse spatial resolution given by the ADC–HBV images and are depicted and listed in Figure 1 (Panel C). The mask images resampled in the axial direction were matched to the resampling and cropping for the T2 images. Shown in Figure 2e is the spatially registered mask derived from Yuan et al.’s segmentation. The zoomed sections highlight the prostate for a particular slice.

### 2.5. Normalized “Green” Image

**Figure 3:**
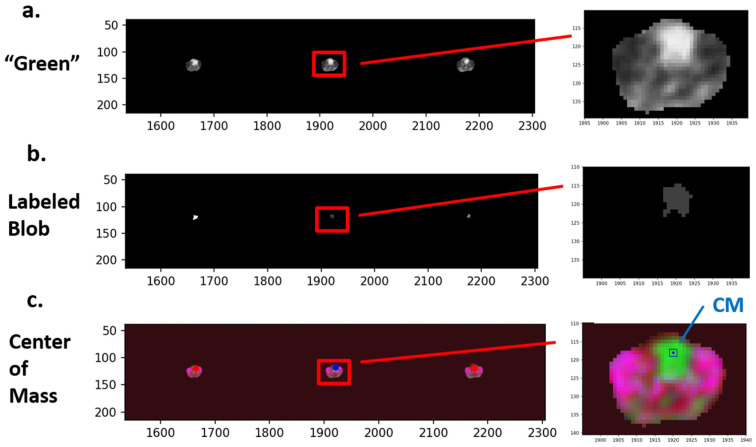
Three consecutive spatially registered slides for “Green” (**a**) grey scale from thresholding the “Green” slides (**b**) blob resulting from labeling the thresholded “Green” slides (**c**) center of mass (CM) (blue box) superimposed onto color composite of stitched hypercube (**c**). Also shown are zoomed regions for the prostate, blob, CM on color composite.

Prostate tumors generally show reduced diffusion rates due to the tumors’ higher cellular density relative to a normal prostate [34] that impedes proton flow. In multi- and bi-parametric MRI prostate cancer imaging, the impeded motion (tumors) is expected to show a lower ADC, a higher HBV and a lower T2 signal intensity relative to normal tissue. Tumors appear green by assigning red, green, and blue colors to ADC, HBV and T2 images, respectively. In this hypothesis, quantifying the “greenness” of a voxel reveals a tumor presence within a prostate that also can be used to sort the blobs based on tumor aggressiveness. An example is shown in Figure 3a. This example was taken from patient #10085 in the PI-CAI data collection.

The “greenness” was “quantified” and is depicted and listed in Figure 1 (Panel D). This study generated a normalized green image by adhering to the following procedure [31]. After the bi-parametric MRI was spatially registered, a prostate mask generated by AI [34] marked the entire prostate (Figure 2e). For voxels residing within the prostate mask, a cumulative histogram for prostate voxel values in each sequence (ADC, high B-value, and T2) determined the 1st-percentile and 98th-percentile values. Possible non-linear effects are minimized because the scanned region is confined to the central part of the image, thereby reducing magnetic inhomogeneities and anomalies. In addition, the analysis is confined to a single organ (prostate) and does not include bony, soft tissue and air. The voxels were linearly scaled based on the 1% and 98% values, and anomalously valued voxels (<1% and >98%) were prevented from distorting the normalization process. The “green” image was formed by computing the ratio for each normal prostate voxel: Green/(Red + Green + Blue) or High B/(ADC + HBV + T2). Figure 3a shows three consecutive “green” slices along with a zoomed image of the prostate from a single slice. The most “tumor-like” voxels possessed the largest green values. Figure 3b shows stitched slices and the blob resulting from labeling the thresholded “Green” slides. Figure 3c superimposes center of mass (CM) (blue box) onto stitched color composite. slides. Also shown are zoomed regions for the prostate, blob, CM on color composite. This example was taken from patient #10085 in the PI-CAI data collection.

### 2.6. Blobbing

Blobbing and labeling [35,36,37,38,39] or connected component labeling in computer vision parlance means objectively aggregating neighboring voxels in a binary image and is depicted and listed in Figure 1 (Panel D). In this study, the blobbing was confined to two dimensions (not three dimensions) and within the transverse plane. The binary image was derived from a detection image (in this application, “Green”) that was divided into tumor or normal prostate voxels based on voxel values exceeding the threshold. Each “tumor” voxel interrogated voxels within a given neighborhood (1 voxel away) based on the 8-pixel neighborhood to see if they were also “tumor.” The erosion and dilation (standard image-processing procedures) eliminated stray isolated, noisy voxels. If the “tumor” voxels were connected, the group of voxels was collected and labeled as a member of a blob. Blobs smaller than 10 voxels (~3 × 10^−2^ mL) were filtered out. Figure 3b shows three consecutive blobs derived from the “green” image (Figure 3a), along with a zoomed image of the prostate from a single slice.

### 2.7. Blob Selection

Prostate tumors are often multifocal and occupy spatially distinct regions. The individual tumor grades can vary within a given patient. One option of this study was to autonomously select a single blob from the normalized “green” image (not manually choose the desired blob), which is depicted and listed in Figure 1 (Panel F). A blob’s morphology metrics (see Section 2.11 and Table 2) and values related to “greenness” of a given blob were examined as possible features for selecting the optimal blob that truly characterizes the prostate cancer. Specifically, such features include maximal blob size, weighted (by blob size) eccentricity (see Section 2.11 for eccentricity description), maximum eccentricity, maximal “green” value, minimal “green” value standard deviation, maximal sliding window “green” value (see Section 2.11 for description of the sliding-window procedure), minimal sliding-window standard deviation, maximal sliding-window covariance (average green/standard deviation) signal-to-noise ratio (SNR), and relative blob position in the scan. All blobs for a given patient were rated relative to each other. The relative contributions of the independent variables were determined by a logistic multi-variable fit to the ground-truth CSPCa (from PI-CAI biopsies and evaluations).

In addition, this study computed the average grade over all blobs.

### 2.8. Covariance Matrix Processing

The covariance matrix (CM) quantitatively characterized the background from the entire prostate organ [28,29,30,31,32] and is depicted and listed in Figure 1 (Panel G). The CM’s components included the variances for each component (ADC, HBV, and T2) and the cross-covariances among the components. Accounting for correlation among the components was required to avoid possible overcounting and proper component combination. Whitening the CM transformed [39,40] the components into a totally decorrelated space with unit variation and zero background [40]. The whitened space provided a reference to help quantify the spectral metrics of the tumor. Additional processing was required to reduce noise by removing noisy principal components to put limits on the coefficients and to reduce the effects of spectral anomalies in the background. To handle these issues, this study applied principal component filtering [41], two types of regularization [42], and elliptical volume minimization [43].

### 2.9. Signal-to-Clutter Ratio Computation

The signal-to-clutter ratio is a ratio of how much the tumor spectral signal departs from the normal prostate [28,29,30,31,32,40] and is depicted and listed in Figure 1 (Panel H). This ratio correlated with tumor grade [28,29,30,31,32] and was treated as a feature for predicting CsPCa. The SCR used all three images (ADC, HBV, and T2). The tumor signature was a three-dimensional vector with components of ADC, HBV, and T2 derived from the center of mass of the blob (Figure 3c, where CM denotes center of mass) or the greenest part of the blob. The average normal prostate background was subtracted from the tumor signature and provided a reference. From the background covariance matrix, whitening removed “overcounting” due to correlation among the three images. Further processing of the covariance matrix (as noted above, in Section 2.8) through filtering, regularization, and elliptical volume minimization removed noise, imposed limits on the coefficients and eliminated anomalies to further enhance the SCR calculation.

### 2.10. Adaptive Cosine Estimator (ACE)

The adaptive cosine estimator (ACE) algorithm was applied to the spatially registered BP-MRI [28,29,30] to compute a metric associated with the tumor volume. The ACE is a supervised detector applied to multi- and hyperspectral images [28,29,30]. The ACE is a decision surface bearing some semblance to the SCR, a scalar. The tumor is characterized by a signature vector (three-dimensional components from the ADC, HBV, and T2) that provides the “supervision.” The background from the prostate organ is characterized by a 3 × 3 covariance matrix that computes the variances for all three dimensions and accounts for cross-correlation across all three dimensions. The decision surface is a multi-spectral cone (three-dimensional in this study). Voxels whose values exceeded an ACE threshold (or whose ACE values reside inside the conical decision surface) were assigned to the tumor. Normal tissue was assigned to voxels whose values resided below the ACE threshold. Voxels exceeding the given threshold were summed. This sum was converted to the volume based on the MRI spatial resolution. References [40,41,43] summarize the mathematics behind the tumor volume computation.

### 2.11. Spectral/Morphological Independent Variables

Multi-variable logistic regression predicted the CsPCa [44]. Along with the SCR, morphological characteristics describing the shape and size of the blob and standard clinical metrics were treated as possible independent variables. This study computed all possible combinations of one-, two-, and three-variable regression fits and found the top performers based on the AUC. This procedure is depicted and listed in Figure 1 (Panel J). Table 2 lists the independent variables used to predict clinically significant prostate cancer. The independent variables are separated into those that can be identified as being related to the SCR, morphological, and clinical.

The roster of independent variables included spectral/morphological features such as the blob size, the standard deviation of “green” values in the blob, the position of the blob within the scan, the global and sliding-window covariance, the z-score or the relative size of the maximum green value to the average prostate value, and is depicted and listed in Figure 1 (Panel I).

**Figure 4:**
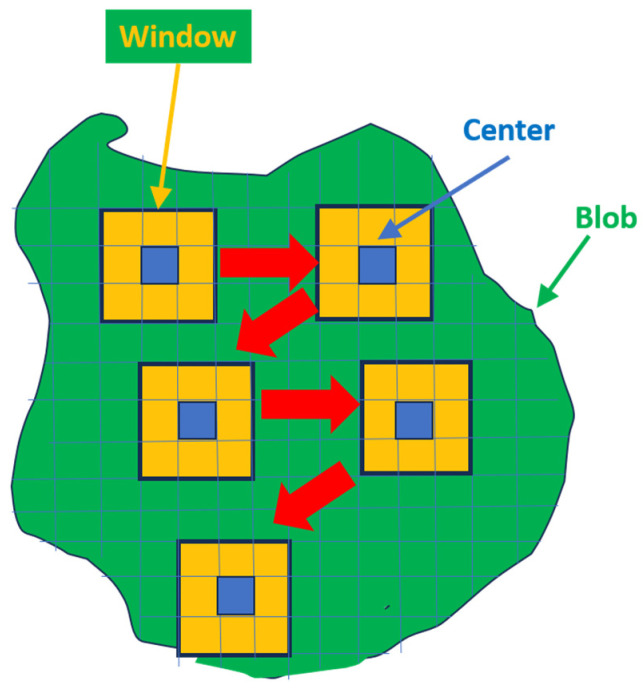
Schematic of sliding window as the 3 × 3 window moves across the blob. Red arrows schematically shows sliding window translation across blob.

The features related to the SCR are listed under the SCR column in Table 2. The list includes the entire gamut of processes used to handle the covariance matrix for the prostate. Specifically, the regularization (Reg), modified regularization (Mod Reg), principal component filtering (PC Filter), elliptical volume minimization (Ellip Min), z-score, and unprocessed. In addition, the SCR values are listed in linear or in decibels (dB) in order to account for possible non-linear relationship between SCR and the CsPCa. In addition, the position of the largest blob relative to the center of the scan in the axial direction, the covariance (Maximum Green/Blob volume), listed as CV and Slide CV (derived from sliding windows; see below for discussion and Figure 4), z-score, standard deviation for the largest blob (Std Dev), and signal-to-noise ratio (SNR) of the blob.

The morphology column lists candidate variables related to the size of the blob and its shape or eccentricity. These morphological features originated from spectral signatures or the spatially registered dataset.

The eccentricity [28,29,30] was computed for every labeled blob. For a given blob k, the moment of inertia matrix I was computed. Next, the eigen equation (using the moment of inertia I) and the two eigenvalues were computed for each blob. The largest eigenvalue was assigned to the large axis l_k_, and the smaller eigenvalue was assigned to the transverse moment s_k_. The eccentricity E_k_ for the kth blob is the normalized difference between the major axis l_k_ and minor axis s_k_. The eccentricity values E_k_ range from 0 (spherical shape) to 1 (line). References [28,29,30] offer a more detailed summary of the eccentricity computation, along with clarifying equations.

To help account for a tumor’s heterogeneity, a “sliding window” (Figure 4) was applied across the blob [31] in the transverse section (two dimensions). Red arrows schematically shows sliding window translation across the blob. Specifically, a sliding window (3 × 3 voxels) moved one voxel at a time across the blob, computing, recording and storing the average and standard deviation for each window. This procedure was replicated and extended over the entire blob. The largest “green” and largest covariance (average/standard deviation) were noted. The sliding-window-derived features are prefaced as “Slide” in the “Morphology” column in Table 2.

Standard clinical-based features (read from the meta/clinical data accompanying the MRI), namely, prostate serum antigen (psa), patient age, prostate volume, and PSAD, which were recorded as part of the patient data in PI-CAI, were added as candidate independent variables in the multi-variable logistic regression fit.

Table 2 shows the “reservoir” of the 16 variables used in the logistic regression fit. All combinations of these variables were calculated using a single variable (16 options), two variables (120 options), and three variables (560 options) in the logistic regression fit. The tables in the Results section (Section 3) show only the combinations that achieved the top four highest AUCs.

### 2.12. CsPCa Prediction and Assessment Metrics

Liblinear solver was applied to the optimization problem for this study’s logistic regression [44]. Liblinear is based on a linear algorithm that uses a coordinate descent method. This solver is efficient for small to medium-sized datasets.

The CsPCa for each patient was assessed by radiologists and pathologists using visual evaluation of MRI and pathology and provided the “ground truth” to help evaluate the algorithms. This procedure is depicted and listed in Figure 1 (Panel K).

Standard cross-validation procedures were applied to advance model performance and reliability, adapted to predict clinical outcomes for the 76-patient cohort. The patients were randomly assigned to five folds using stratified k-folding to reduce the imbalance in the training sets. The training and logistic regression coefficients were determined from patients in 4 folds, and the fitted coefficients were then applied to the 5th fold, and the performance metrics were recorded [45], specifically the mean area under the curve (AUC) and the associated 95% confidence interval. In addition, to test for possible imbalances in training sets, the precision, recall, and F1-score (F1) were also computed, along with their confidence intervals. The same procedure was replicated and applied to all training–test fold configurations. This procedure is depicted and listed in Figure 1 (Panel L).

## 3. Results

The AUC computed from the receiver operating curve (ROC) characterized the accuracy for predicting a patient’s prostate cancer was clinically significant against the probability of false positives. The number of variables in the logistic regression varied from one to three. The algorithm performance was noted for all patients as well as those patients with blobs exceeding a certain minimum value. This study assessed the performance of selecting a given blob against averaging over all blobs.

Table 3 summarizes the range in times (minutes) to process individual tasks using intervention-required methods and automated approaches to compute CsPCa. The times were recorded by noting the processing times for individual patients. Processing times for individual patients can vary due to differences in individual patient prostate volumes and the number of axial slices. Table 3 divides the processes into spatial registration, choosing a signature, outlining the prostate, and analyzing and predicting the CsPCa. Automation expedites the calculation for registration, signature identification, and prostate outline for each individual patient. It should be noted that the analysis time for the intervention and automated process is the same (unlike the other tasks). However, automating the analysis greatly expedited the calculation for 10 patient files. The processing times for a single patient and 10 patients are, therefore, separately listed. The last row lists the total computation times. The last column lists the time saved by automating the process listed in the process column. The total savings in time by automating the process ranged from 240 to 350 min for 10 patients. Applying the new automation tools alone reduced the processing time by 80 to 170 min for 10 patients. After automating all the listed processes, the calculation time for 10 patients was roughly 40 to 80 min, permitting ultimate calculation for 1000 patients.

**Figure 5:**
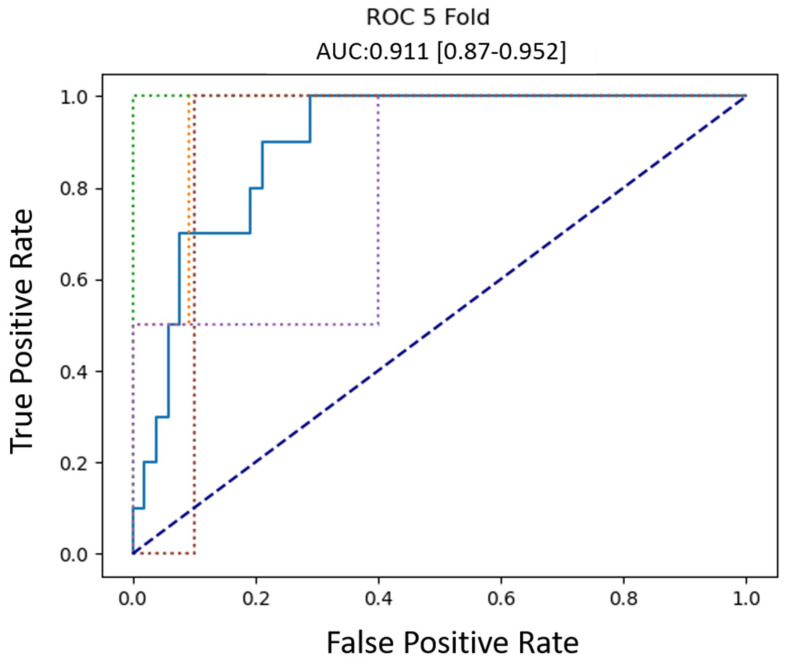
A sample ROC curve from logistic regression using three variables (SCR selected z-score, patient age, and prostate volume). Solid line shows average for 5 folds. The dotted lines show the ROC curves for the five individual folds with AUCs of 0.955, 1.0, 0.90, 0.80, and 0.90.

Figure 5 shows an ROC curve displaying the probability of correctly assessing patients’ clinical status versus the probability of a false positive. This particular calculation evaluated the logistic fit of three variables, selected SCR (dB) registration, psa, and prostate volume, to the CsPCa. The solid line shows the ROC curve with the AUC average over five folds of 0.911 and a 95% confidence interval from 0.870 to 0.952, and the dotted lines show the ROC curves for the five individual folds with AUCs of 0.955, 1.0, 0.90, 0.80, and 0.90.

Table 4, Table 5, Table 6 and Table 7 show the computed AUC, precision, recall, F1-score and 95% confidence intervals from five-fold logistic regression fitting involving one, two, and three variables. Logistic regression was applied to all combinations of variables, but only the top four scorers are shown in Table 4, Table 5, Table 6 and Table 7. Table 4 shows the AUC, precision, recall, and F1-score from selecting a single blob for a given patient but restricting the analysis to patients exceeding a threshold blob size (>0.03 cm^2^). Table 5 summarizes the data without a volume threshold. Table 6 displays data using the average blob SCR for a given patient and after applying the volume threshold. Table 7 summarizes the results from averaging the SCR from all blobs within a patient and no volume threshold.

Several conclusions can be drawn from the results summarized in the tables. Generally, better accuracy and a larger AUC result from using more variables. In one typical example (Table 4, after removing smaller blobs), the best mean AUCs are 0.845, 0.891, and 0. 911 for one, two, and three variables, respectively. Similarly, Table 5, Table 6 and Table 7 show better mean AUCs by increasing the number of variables in the logistic fit to CsPCa. In addition, higher AUCs are achieved for combinations of variables that are less correlated with each other, such as combining the SCR with blob morphology features or combining it with clinical metrics, as seen in the four largest mean AUCs in Table 4, Table 5, Table 6 and Table 7. As an example, the highest mean AUC in Table 4 is achieved by combining the disparate independent variables SCR Select Reg, prostate volume, and psa (a clinical variable).

Table 4 and Table 6 also summarize calculations restricted to blobs exceeding 0.1 cm^3^. For patients with autonomously selected target signatures, filtering out patients whose blobs exceed 0.1 cm^3^ results in a larger AUC (for three variables, 0.911 in Table 4 vs. 0.792 in Table 5). For the averaged blobs, removing small blobs results in a comparable AUC (for three variables, 0.861 in Table 6 vs. 0.792 in Table 7). Selecting blobs rather than averaging over all blobs also results in a larger AUC.

The high precision, recall, and F1-score (F1) showed that any imbalances in training sets were mitigated by stratified folding.

## 4. Discussion

This retrospective pilot study developed, applied, and tested new automation tools to (1) assemble spatially registered and stitched hypercubes composed of bi-parametric MRI of patients with prostate cancer, (2) find areas of potential cancer and generate spectral and related morphological features, and (3) use those spectral-related features to predict whether patients have clinically significant prostate cancer. This study demonstrated the feasibility of applying these spectral/statistical algorithms with minimal training that achieve comparable accuracy for determining the clinical significance of prostate cancer with AI and demonstrated greater processing speed using the new automation tools and supports the use of this approach for future large studies.

High-achieving AI algorithms that participated in the analyses of the prostate bp-MRI for the PI-CAI Challenge [23,24] provided a benchmark and part of the external validation for this study. PI-CAI gathered and provided public access to BP-MRI scans of 1500 prostate patients’ exams. PI-CAI also included clinical and acquisition variables in order to test and validate AI algorithms’ performance in CsPCa detection and diagnosis. The PI-CAI challenge design followed the recommendations from a Scientific Advisory Board to ensure meaningful validation of prostate-AI assessments for ultimate translation to the clinic. A high-achieving algorithm applied to large number of patient AI studies from the PI-CAI Challenge [23,32] found a mean AUC of 0·91 [95% CI: 0.87–0.94] vs. the present simpler spectral/statistics study (patient number: 76), which found mean AUC = 0.911 [95% CI: 0.877–0.952] after screening for low-volume tumors and 0.792 [0.656–0.928] without screening for blob size. In addition, a previous direct 42-patient-to-patient study [32] compared Yuan’s high-achieving algorithm (second best out of 293 competitors) in the Challenge with spectral/statistics using probability (no threshold application) of CsPCa logistically fit to CsPCa AUC = 0.503 vs. the best two-feature-fit spectral/statistics of AUC = 0.695.

The total savings in time by automating the calculation ranged from 240 to 350 min for 10 patients. The additional total savings in time by applying the new automation tools reduced the processing time by 80 to 170 min for 10 patients. After full automation, the overall processing time for 10 patients was roughly 40 to 80 min. It should be noted that spectral/statistics procedures require minimal training and computations and a limited number of variables.

This retrospective pilot study found certain limits for future efforts. Employing a greater number of variables for the logistic regression fits achieved better prediction accuracy and larger AUC results. In addition, higher AUCs were achieved for combinations of variables that were less correlated with each other, such as combining the SCR with blob morphological features or with clinical metrics. Removing patients whose blobs were smaller than 0.03 cm^3^ resulted in larger AUCs. Patients with smaller blob volumes have been found to have lower ISUP grades [28,29,30]. Excluding patients with below-threshold tumor volumes is unlikely to result in missed detection of aggressive tumors or in over-treatment. Selecting blobs rather than averaging over all blobs also results in a larger AUC. Optimal selection of blobs requires further investigation.

The present study used an AI algorithm to segment the normal prostate. However, there are a number of segmentation algorithms for multi-spectral images that do not employ AI, such as k-means [46,47], which can also be used in future efforts. The AI approach was used in the present study primarily to expedite the analysis for the relatively large patient cohort and for immediate analysis of greater than 1000 patients. However, future efforts will incorporate the well-studied and established non-AI segmentation method, K-means, into the processing.

Using only spectral/statistical approaches requires minimal training relative to AI [28,29,30,31,32]. This simplification will presumably reduce the burden on environmental and energy resources and extend application of quantification and accuracy for determining the clinical status of a tumor. At worst, the spectral features that are uncorrelated with AI features can be added to the AI-derived features and boost the AI/spectral accuracy. Automating the spectral/statistical approach will aid in analyses for large studies and protocols by speeding up the process. Nevertheless, “semi-manual” generation and manipulation of spatially registered hypercubes and spectral signatures is simple enough for handling a single patient in the clinic.

The present study of 76 patients processed with autonomous procedures found slightly poorer performance than those found in an earlier 42-patient study [30,31,32] that required greater interventions. This may be due to a different patient set and remains under investigation. As part of the investigation into the disparity in performance between the 42-patient study and this 76-patient examination, this study examined alternative approaches for spatially registering the MP-MRI, specifically, re-slicing the ADC–HBV axial slices to match the T2 slices. The present analysis applied weighted averaging of ADC–HBV slices based on the relative distance, as denoted by the positions listed in the skins from the T2 axial positions denoted in the scan. Such an approach is expected to more closely match the ADC–HBV slices to the T2 slices. However, averaging may introduce spatial artifacts, especially near the edges of the prostate, and it may also add noise. The other approach chose the ADC–HBV position that was closest to the T2 position without averaging. Both spatial registration approaches achieved similar results in terms of AUC calculations.

The patients within the PI-CAI data collection were scanned in five different centers using a variety of scanning and setup techniques. It was, therefore, difficult to develop a general spatial registration algorithm that could handle all clinical situations. As a result, this study employed a check of the spatial registration and appropriate small adjustments by translating the ADC–HBV slices in the axial and transverse directions.

The goal guiding this retrospective pilot study is to test techniques for future tests on larger numbers of patients. Like all pilot studies, testing this approach among a larger number of patients is essential in order to validate these findings. The small samples lack statistical power, although the computed results and confidence intervals suggest this approach shows promise. The goal of this pilot study is to refine techniques, not to definitively prove the hypothesis. Pilot studies often test instruments, procedures, approaches, sampling, and protocols in early stages. These provisional designs are prone to bias, measurement error, and inconsistency. However, this study attempted to avoid bias by examining consecutive patients rather than deliberately selecting patients and to avoid misrepresentation of the wider population, which weakens generalizability.

## 5. Conclusions

This study developed, applied, and tested the automation of most components for hyperspectral cube formation and processed results to expedite accurate prediction of clinically significant prostate cancer. This retrospective pilot work demonstrates the feasibility of a novel approach that can be applied to analyze a large patient cohort. This study also examined potential limits, such as the role that minimal blob size plays in predicting CsPCa, for future studies.

## Figures and Tables

**Table 1:** Summary of clinical data for 76 patients; analysis within clinically significant and insignificant means.

Clinical	Insignificant Mean [95% CI]	Significant Mean [95% CI]	*p*-Value	All Mean [95% CI]
Patient age (years)	63.74 [61.93, 65.56]	65.64 [60.60, 70.68]	0.458	64.09 [62.39, 65.79]
PSA (ng/mL)	10.12 [7.44, 12.81]	18.67 [8.48, 28.86]	0.101	11.63 [8.81, 14.44]
Prostate volume (mL)	69.67 [58.85, 80.50]	63.15 [34.90, 91.41]	0.649	68.53 [58.61, 78.45]
PSAD (ng/mL/mL)	0.259 [0.156, 0.361]	0.198 [0.120, 0.276]	0.346	0.248 [0.125, 0.372]

**Table 2:** List of independent variables.

SCR	Morphology	Clinic
SCR (dB) Select Reg	Size	patient_age
SCR (dB) Select Mod Reg	Volume (best) (cc)	PSA
SCR (dB) Select PC filter	Max Blob Volume (cc)	prostate_volume
SCR (dB) Select Elliptical	Mean Blob Volume (cc)	PSAD
SCR (dB) Select z score	Total Volume (cc)	
SCR (dB) Select Unprocessed	Weighted Eccentricity	

**Table 3:** Processing time ranges (minutes) for interventions required and automation.

Process	Intervention (1 File)	Intervention (10 Files)	Automation (1 File)	Automation (10 Files)	Time Savings (10 Files)
Registration, stitching	15–20	150–200	1–2	10–20	130–190
Choosing signature	5–10	50–100	1–2	10–20	30–90
Prostate outline	10–15	100–150	1–2	10–20	80–140
Analysis	1–2	20–40	1–2	10–20	0–30
Total	31–47	220–490	4–8	40–80	240–350

**Table 4:** Selected blobs, mean AUC, precision, recall, F1-score and [confidence interval] for 1, 2, and 3 variables with small blobs removed. Highest 4 mean AUCs.

Configuration (1 Variable, Remove Small Blob)	AUC [95% CI]	Precision [95% CI]	Recall [95% CI]	F1-Score [95% CI]
SCR (dB) Select Reg	0.845 [0.79–0.9]	0.832 [0.824–0.84]	0.96 [0.93–0.99]	0.891 [0.874–0.908]
SCR (dB) Select Mod Reg	0.835 [0.772–0.898]	0.832 [0.824–0.84]	0.96 [0.93–0.99]	0.891 [0.874–0.908]
SCR (dB) z-Score	0.793 [0.653–0.933]	0.852 [0.837–0.867]	0.98 [0.955–1.00]	0.91 [0.906–0.914]
SCR (dB) Select Unprocessed	0.775 [0.694–0.856]	0.832 [0.824–0.84]	0.96 [0.93–0.99]	0.891 [0.874–0.908]
**Configuration (2 Variables, Remove Small Blobs)**				
SCR (dB) Select Reg, PSAD	0.891 [0.837–0.945]	0.861 [0.832–0.89]	0.96 [0.93–0.99]	0.907 [0.879–0.935]
SCR (dB) Select Mod Reg, PSAD	0.882 [0.831–0.933]	0.861 [0.832–0.89]	0.96 [0.93–0.99]	0.907 [0.879–0.935]
SCR (dB) Select Reg, psa	0.863 [0.809–0.917]	0.864 [0.837–0.891]	0.98 [0.955–1.00]	0.918 [0.895–0.941]
SCR (dB) Select Reg, Prostate Volume	0.855 [0.803–0.907]	0.835 [0.829–0.841]	0.98 [0.955–1.00]	0.902 [0.888–0.916]
**Configuration (3 Variables, Remove Small Blobs)**				
SCR (dB) Select Reg, psa, Prostate Volume	0.911 [0.87–0.952]	0.861 [0.832–0.89]	0.96 [0.93–0.99]	0.907 [0.879–0.935]
SCR (dB) Select Unprocessed, Total Volume, PSAD	0.901 [0.849–0.953]	0.847 [0.824–0.87]	0.96 [0.93–0.99]	0.899 [0.875–0.923]
SCR (dB) Select Reg, Total Volume, PSAD	0.901 [0.857–0.94]	0.847 [0.824–0.87]	0.96 [0.93–0.99]	0.899 [0.875–0.923]
SCR (dB) Select Reg, Max Blob Volume, PSAD	0.9 [0.848–0.952]	0.847 [0.824–0.87]	0.96 [0.93–0.99]	0.899 [0.875–0.923]

**Table 5:** Selected blobs, mean AUC, precision, recall, F1-score and [confidence interval] for 1, 2, and 3 variables. Highest 4 mean AUCs.

Configuration (1 Variable)	AUC [95% CI]	Precision [95% CI]	Recall [95% CI]	F1-Score [95% CI]
PSAD	0.749 [0.642–0.856]	0.836 [0.82–0.852]	0.967 [0.942–0.992]	0.896 [0.884–0.908]
psa	0.69 [0.611–0.769]	0.836 [0.82–0.852]	0.967 [0.926–1.00]	0.895 [0.873–0.917]
SCR (dB) Select Reg	0.618 [0.491–0.745]	0.806 [0.79–0.822]	1.0 [1.0–1.0]	0.892 [0.882–0.902]
SCR (dB) Total Volume	0.617 [0.498–0.736]	0.806 [0.79–0.822]	1.0 [1.0–1.0]	0.892 [0.882–0.902]
**Configuration (2 Variables)**				
Total Blob Volume, PSAD	0.765 [0.671–0.859]	0.836 [0.82–0.852]	0.967 [0.942–0.992]	0.896 [0.884–0.908]
Total Blob Volume, psa	0.764 [0.665–0.863]	0.838 [0.821–0.855]	0.983 [0.962–1.00]	0.905 [0.892–0.918]
Max Blob Volume, PSAD	0.753 [0.671–0.835]	0.836 [0.82–0.852]	0.967 [0.942–0.992]	0.896 [0.884–0.908]
Prostate Volume, PSAD	0.742 [0.65–0.834]	0.836 [0.82–0.852]	0.967 [0.942–0.992]	0.896 [0.884–0.908]
**Configuration (3 Variables)**				
Max Blob Volume, Mean Blob Volume, PSAD	0.792 [0.656–0.928]	0.836 [0.82–0.852]	0.967 [0.942–0.992]	0.896 [0.884–0.908]
Total Blob Volume, Mean Blob Volume, PSAD	0.768 [0.652–0.884]	0.836 [0.82–0.852]	0.967 [0.942–0.992]	0.896 [0.884–0.908]
SCR (dB) Select Unprocessed, Total Volume, PSAD	0.765 [0.683–0.847]	0.825 [0.809–0.841]	0.967 [0.942–0.992]	0.889 [0.88–0.898]
Total Volume, Max Blob Volume, psa	0.765 [0.665–0.865]	0.838 [0.821–0.855]	0.983 [0.962–1.004]	0.905 [0.892–0.918]

**Table 6:** Average blob chosen. Mean AUC, precision, recall, F1-score and associated [confidence interval] for 1, 2, and 3 variables with small blobs removed. Highest 4 mean AUCs.

Configuration (1 Variable, Remove Small Blob)	AUC [95% CI]	Precision [95% CI]	Recall [95% CI]	F1-Score [95% CI]
psa	0.764 [0.658–0.87]	0.867 [0.842–0.892]	1.0 [1.0–1.0]	0.928 [0.914–0.942]
PSAD	0.751 [0.616–0.886]	0.862 [0.836–0.888]	0.962 [0.933–0.991]	0.908 [0.889–0.927]
SCR (dB)Ave z-Score	0.699 [0.597–0.801]	0.867 [0.848–0.886]	0.98 [0.955–1.005]	0.919 [0.908–0.93]
SCR (dB) Ave Reg	0.65 [0.555–0.745]	0.838 [0.834–0.842]	1.0 [1.0–1.0]	0.912 [0.91–0.914]
**Configuration (2 Variables, Remove Small Blobs)**				
Total Blob Volume, psa	0.836 [0.782–0.89]	0.864 [0.837–0.891]	0.98 [0.955–1.00]	0.918 [0.895–0.941]
Prostate Volume, psa	0.821 [0.732–0.910]	0.864 [0.837–0.891]	0.98 [0.955–1.00]	0.918 [0.895–0.941]
Total Blob Volume, PSAD	0.815 [0.756–0.874]	0.864 [0.837–0.891]	0.98 [0.955–1.00]	0.918 [0.895–0.941]
Max Blob Volume, psa	0.805 [0.745–0.865]	0.864 [0.837–0.891]	0.98 [0.955–1.00]	0.918 [0.895–0.941]
**Configuration (3 Variables, Remove Small Blobs)**				
Total Blob Volume, Wtd Eccentricity, PSAD	0.861 [0.797–0.925]	0.864 [0.837–0.891]	0.98 [0.955–1.00]	0.918 [0.895–0.941]
Max Blob Volume, Prostate Volume, psa	0.861 [0.797–0.925]	0.864 [0.837–0.891]	0.98 [0.955–1.005]	0.918 [0.895–0.941]
SCR (dB) Ave z-Score, Max Blob Volume, psa	0.848 [0.763–0.933]	0.897 [0.859–0.935]	0.98 [0.955–1.00]	0.935 [0.911–0.959]
SCR (dB) Ave z-Score, Max Blob Volume, PSAD	0.838 [0.754–0.922]	0.894 [0.854–0.934]	0.96 [0.93–0.99]	0.925 [0.895–0.955]

**Table 7:** Average blob chosen. Mean AUC, precision, recall, F1-score and associated [confidence interval] for 1, 2, and 3 variables. Highest 4 mean AUCs.

Configuration (1 Variable)	AUC [95% CI]	Precision [95% CI]	Recall [95% CI]	F1-Score [95% CI]
PSAD	0.749 [0.642–0.856]	0.836 [0.82–0.852]	0.967 [0.942–0.992]	0.896 [0.884–0.908]
psa	0.69 [0.611–0.769]	0.836 [0.82–0.852]	0.967 [0.926–1.00]	0.895 [0.873–0.917]
Total Blob Volume	0.617 [0.498–0.736]	0.806 [0.79–0.822]	1.0 [1.0–1.0]	0.892 [0.882–0.902]
SCR (dB) Ave z-Score	0.613 [0.405–0.821]	0.842 [0.817–0.867]	0.982 [0.959–1.00]	0.905 [0.896–0.914]
**Configuration (2 Variables)**				
Total Blob Volume, PSAD	0.765 [0.671–0.859]	0.836 [0.82–0.852]	0.967 [0.942–0.992]	0.896 [0.884–0.908]
Total Blob Volume, psa	0.764 [0.665–0.863]	0.838 [0.821–0.855]	0.983 [0.962–1.00]	0.905 [0.892–0.918]
Max Blob Volume, PSAD	0.753 [0.671–0.835]	0.836 [0.82–0.852]	0.967 [0.942–0.992]	0.896 [0.884–0.908]
Prostate Volume, PSAD	0.742 [0.65–0.834]	0.836 [0.82–0.852]	0.967 [0.942–0.992]	0.896 [0.884–0.908]
**Configuration (3 Variables)**				
Max Blob Volume, Mean Blob Volume, PSAD	0.792 [0.656–0.928]	0.836 [0.82–0.852]	0.967 [0.942–0.992]	0.896 [0.884–0.908]
SCR (dB) Ave Reg, Total Blob Volume, psa	0.784 [0.668–0.9]	0.838 [0.821–0.855]	0.983 [0.962–1.00]	0.905 [0.892–0.918]
SCR (dB) Ave Unprocessed, Total Volume, PSAD	0.776 [0.682–0.87]	0.836 [0.82–0.852]	0.967 [0.942–0.992]	0.896 [0.884–0.908]
Total Volume, Mean Blob Volume, PSAD	0.768 [0.652–0.884]	0.836 [0.82–0.852]	0.967 [0.942–0.992]	0.896 [0.884–0.908]

## Data Availability

The dataset: https://pi-cai.grand-challenge.org/; accessed on 3 March 2023.

## Abbreviations

The following abbreviations are used in this manuscript:

SCR	Signal-to-clutter ratio
PI-CAI	Prostate cancer artificial intelligence
AUC	Area under the curve
F1	F1-score
ROC	Receiver operating characteristic
MP-MRI	Multi-parametric MRI
ADC	Apparent diffusion coefficient
HBV	High B-value
psa	Prostate serum antigen
PSAD	Prostate serum antigen sensitivity
ACE	Adaptive cosine estimator
BP-MRI	Bi-parametric MRI
CsPCa	Clinically significant prostate cancer
DWI	Diffusion weighted imaging
Mod Reg	Modified regularization
Reg	Regularization
PC	Principal component
DL	Deep learning
AI	Artificial intelligence
SNR	Signal-to-noise ratio
ISUP	International Society Urological Pathology
PI-RADS	Prostate Imaging–Reporting and Data System
CV	Covariance
